# Greenway interventions effectively enhance physical activity levels—A systematic review with meta-analysis

**DOI:** 10.3389/fpubh.2023.1268502

**Published:** 2023-12-06

**Authors:** Yujia Deng, Jinghong Liang, Qibing Chen

**Affiliations:** ^1^College of Landscape Architecture, Sichuan Agricultural University, Chengdu, China; ^2^Department of Maternal and Child Health, School of Public Health, Sun Yat-sen University, Guangzhou, China

**Keywords:** greenway, physical activity, active travel, environment, meta-analysis

## Abstract

**Background:**

Previous studies have examined the impact of greenway interventions on physical activity (PA); however, the results have been inconclusive. In order to address this issue, our study conducted a systematic review with meta-analysis to thoroughly evaluate the evidence and determine the effectiveness of greenway interventions in promoting PA.

**Methods:**

We conducted a comprehensive search of literature databases, such as Web of Science, EMBASE, PubMed (via Medline), Cochrane Library, and Scopus, up to June 15, 2023. To synthesize the available evidence, we performed a meta-analysis using a random effects model. The quality of the included studies was assessed using the criteria developed by the Agency for Healthcare Research and Quality and the Newcastle-Ottawa Scale.

**Results:**

A total of 9 publications were identified, involving 6, 589 individuals. The overall quality of most included studies was rated as moderate to high. Our study found that the greenway was effective in promoting PA among participants. Specifically, active travel (AT) showed a standard mean difference (SMD) of 0.10 [95% confidence interval (CI): 0.04 to 0.17], moderate-to-vigorous PA had an SMD of 0.11 (95% CI: 0.02 to 0.20), and total PA had an SMD of 0.14 (95% CI: 0.06 to 0.21). We also observed significant differences in AT levels among participants based on greenway characteristics, exposure distance, exposure duration, and male-to-female ratio.

**Discussion:**

Newly developed or upgraded greenways have been shown to effectively promote PA. Additionally, research suggests that the longer a greenway has been in existence, the greater the benefits it provides for PA. As a result, the construction of greenways should be recognized as an effective public health intervention.

## 1 Introduction

The WHO defines physical activity (PA) as any movement of the body that requires energy expenditure and is produced by skeletal muscles (1). PA not only improves physical health but also enhances mental and social wellbeing (2). According to the latest global estimates, more than 80.0% of adolescents and 27.0% of adults fail to meet the recommended levels of PA set by the WHO (3, 4). Moreover, the COVID-19 pandemic has led to an increase in sedentary behavior (5), exacerbating the already prevalent issue of insufficient PA (6). Physical inactivity is a significant public health concern that affects individuals throughout their lives and imposes a substantial socioeconomic burden.

The built environment is a significant factor in determining PA levels (7). Multiple studies have consistently shown a positive association between the built environment and PA. It is particularly noteworthy that interventions that focus on improving pedestrian and bicycle transportation, as well as land use and environmental design, have been successful in promoting PA (7, 8). This highlights the importance of creating environments that encourage an active lifestyle. Creating PA-friendly built environments should be prioritized in the field of international health. Specifically, the development of green spaces, such as greenways and parks, within the built environment, is considered an intervention that has the potential to increase PA levels among both children and adults. These green spaces can provide attractive surroundings, easy accessibility, opportunities for social interaction, stress reduction, and essential amenities and infrastructure (9). In recent years, there has been an increasing number of studies examining the relationship between greenways and PA, but the results have been inconsistent. Xie et al. (10) conducted a study that showed a positive influence of a large-scale greenway on both moderate-to-vigorous PA (MVPA) and overall PA. However, Burbidge and Goulias (11) discovered an unexpectedly negative impact on both total PA and walking frequency 5 months after the trail's construction. Moreover, West and Shores (12) found no significant differences in walking, moderate activity, or vigorous activity between the experimental and control groups before and after the greenway's construction. Given the conflicting results, additional research is required to determine the impact of greenway interventions on PA. Furthermore, only four articles have conducted systematic reviews on the relationship between green space interventions (such as greenways, parks, and similar interventions) and PA, and they have reported promising findings (9, 13–15). However, the studies examining greenway interventions included in these reviews are limited to European and American countries, and there is a lack of quantitative evidence. With the significant increase in published research on greenways and their impact on PA, especially including studies conducted in Asian countries and several notable cohort studies published since 2019 (10, 16–18), an updated and comprehensive approach is necessary. The meta-analytic approach offers a statistically robust and objective method of combining diverse empirical findings, expanding the generalizability and significance of conclusions beyond the constraints of individual studies (19). Thus, our aim, through a systematic review with meta-analysis, was to quantify the association between greenways and PA, thereby offering valuable insights for both future academic research and policy-making.

## 2 Methods

This meta-analysis was conducted in accordance with the Cochrane Collaboration Handbook recommendations (20). The article adheres to the PRISMA reporting checklist (21). The analyses were based on previously published studies, thus ethical approval or patient consent was not required.

### 2.1 Search strategies and study selection

An exhaustive literature search was conducted to investigate the relationship between greenways and PA. The search was conducted without any language or publication date restrictions and included relevant studies from the inception of each database up to June 15, 2023. The databases used for the search included Web of Science, EMBASE, PubMed (via Medline), Scopus, and the Cochrane Library. The search was comprehensive and involved combining medical subject headings (MeSH), “Emtree” index terms, and free words using Boolean logic operators. The search terms used encompassed “physical exertion,” “motions,” “walking,” “bicycling,” “greenway,” and “greenways,” among others. The complete search strategy is provided in Appendix 1.

To identify additional potentially relevant studies, we employed a comprehensive search strategy. This involved manually searching the reference lists of relevant published studies, screening top journals in the research area (e.g., Landscape and Urban Planning, Transportation Research Part D: Transport and Environment), reviewing gray literature, and examining significant international academic proceedings. The titles and abstracts retrieved from the initial search were efficiently managed using NoteExpress 3.2 (Aegean Sea Software, Beijing, China). The literature screening process was conducted independently by two researchers, with any discrepancies resolved with the assistance of a third researcher. Duplicate studies were automatically excluded using software functions, and studies unrelated to greenways and PA were removed based on their titles and abstracts. The full texts of the remaining relevant studies were obtained and further screened based on inclusion and exclusion criteria. Finally, the selected citations were cross-validated by the two independent researchers to ensure the inclusion of eligible studies. Throughout the screening process, a third researcher provided supervision.

### 2.2 Eligibility criteria

The studies included in the analysis were required to meet the following criteria.

#### 2.2.1 Populations

The study population consisted of individuals aged 16 and above, residing at different distances from the greenway. There were no restrictions based on gender, health status, or nationality. A sampling process, which involved multiple stages and either stratification, systematic selection, or random selection, was employed to choose the study participants.

#### 2.2.2 Interventions

We included studies that evaluated the impact of developing or upgrading greenways on PA levels of individuals living near these areas. The interventions involved converting existing roads or trails into greenways, which consisted of a combination of bicycle paths and walking trails. These greenways also provided convenient facilities and appealing landscapes (10). Additionally, some studies focused on the creation of entirely new greenways, which were added to existing ones along rivers (22).

#### 2.2.3 Outcomes

The primary outcome measurements included active travel (AT), which encompassed walking and cycling. Previous studies have confirmed the benefits of AT in reducing health risks by promoting PA levels (23–25). The secondary outcome measurements consisted of MVPA, which includes both moderate-intensity PA and vigorous-intensity PA performed at a metabolic equivalent of task (MET) >3 (1). Additionally, total PA, representing the cumulative PA over the past seven-day period, was also assessed. Various measurement approaches were utilized to evaluate the change in outcomes from baseline to endpoint.

#### 2.2.4 Study design

Our study consisted of population-based longitudinal research and repeated cross-sectional studies. We excluded studies that met the following conditions: (1) studies with inaccessible full-texts or data; (2) studies with a research design limited to one experimental group, review or narrative articles, study protocols, or qualitative studies; (3) studies that did not provide specific data on the distance from the participant's residence to the greenway; (4) multiple publications from the same study population analyzing data with the same exposures and outcomes during the same time periods.

### 2.3 Data extraction and quality assessment

Two researchers independently collected vital data from each study and recorded it in a pre-designed Excel spreadsheet. The data included information such as authors, year of publication, study design, region, sample size, female ratio, exposure duration or completion date of the greenway, and PA outcome(s). In cases where the required information was not available in the original studies, efforts were made to contact the authors of potential studies and obtain the necessary data.

The researchers chose specific scales according to the study design of the included studies. Cross-sectional studies were evaluated using the Agency for Healthcare Research and Quality (AHRQ) meta-analysis of statistics assessment and review instrument, while cohort studies were assessed using the Newcastle-Ottawa Scale (NOS) (26). The AHRQ meta-analysis instrument consists of 11 items, detailed in Supplementary Table S2 (27). Each item was assigned a binary score of either “1” if it met the criteria or “0” if it did not. The evaluation and classification of article quality were carried out using the specified criteria: low quality (0–3), moderate quality (4–7), and high quality (8–11). Furthermore, the NOS consists of eight items that are divided into three dimensions: selection, comparability, and outcome, as outlined in Supplementary Table S1 (28). Items falling under the selection and outcome categories can receive a maximum of 1 star each, while comparability permits a maximum of 2 stars. Articles that achieved a NOS score of 7 or higher were classified as “high quality,” while those scoring below 7 were considered “low quality.”

### 2.4 Statistical analyses

In the initial stage, we conducted a conventional pairwise meta-analysis for each comparative trial included in the study (20). For numerical variables, we extracted the mean difference (MD) and standard deviation (SD) of the change from baseline. Alternatively, we transformed the variables into a standardized format. Additionally, we collected exposure estimates with significant effects from the included studies or used estimates from other studies that were most comparable. For instance, He et al. (17) examined the impact of proximity to a greenway on walking outcomes. Participants residing at varying distances (0–1 km, 1–2 km, 2–3 km, 3–4 km, and 4–5 km) were included in the study. The results showed a significant increase in walking time among participants living within a 2-kilometer radius of the greenway. For the meta-analysis, the estimates for the exposed group (0–1 km and 1–2 km) were combined separately from the estimates for the unexposed group (2–3 km, 3–4 km, and 4–5 km), taking into account other relevant studies included in this review (10). To capture the final intervention effect of the study, we extracted data for the last follow-up period from the study that reported estimates for two follow-up periods (29). In cases where the exposure period of the greenway intervention was not specified, we calculated the intervention effect by considering the time difference between the opening of the greenway and the last follow-up as the exposure duration.

To ensure a conservative approach, we utilized a random-effects model (30). This allowed us to calculate the standardized mean difference (SMD), pooled effect sizes, and corresponding 95% confidence interval (CI), taking into account the diverse units of measurement used in the study outcome indicators (31). The quantitative pooled analyses were performed using the random effect model and I-V heterogeneity approach (20). I^2^ statistics were utilized to assess statistical heterogeneity, where values of 25, 50, and 75% were considered as indicating mild, moderate, and high heterogeneity respectively. A *P* > 0.1 was considered as indicating non-statistically significant heterogeneity (32). Additionally, potential bias in small studies was evaluated using a comparison-adjusted funnel plot, which examined publication bias, selective reporting, or other biases. The quantitative Egger's test was conducted to identify the presence of *P* < 0.05 (33). Subgroup analyses were conducted to investigate observed heterogeneity and explore statistically significant differences among the studies. The variables of interest included exposure duration (≥12 months and <12 months), male-to-female ratio (≥1 and <1), exposure distance (within 1.00 km from the greenway and 1.00 km-2.00 km from the greenway), greenway characteristics (including blue space and no blue space), total sample size (>450 and ≤ 450), and region (Europe and America, China and Australia). The statistical analyses were performed using version 14.0 of the STATA software.

## 3 Results

### 3.1 Literature selection

**Figure 1:**
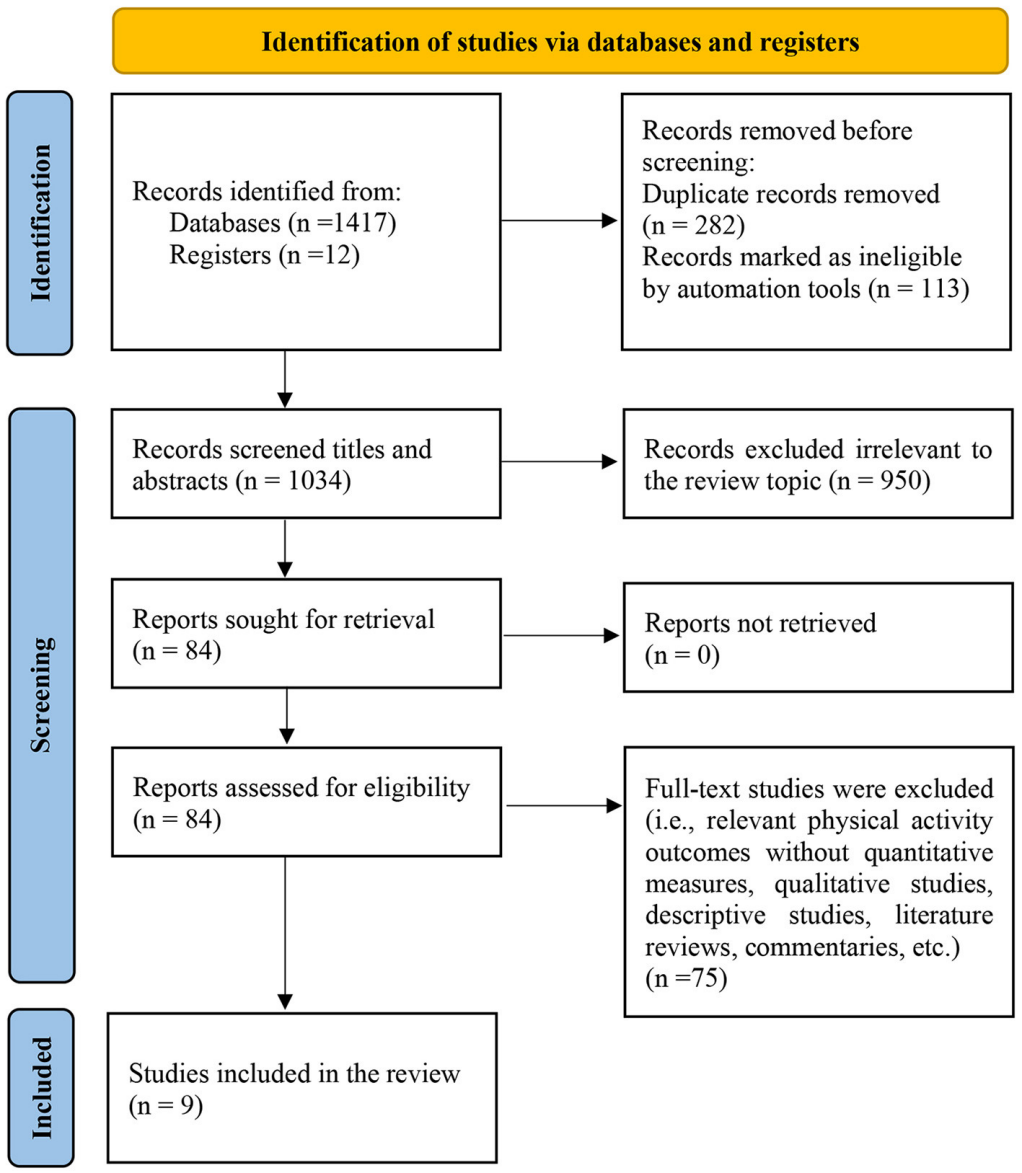
Flowchart of study selection.

In this study, we conducted a comprehensive search across various databases and sources, resulting in a total of 1, 429 publications (Figure 1). After removing duplicate articles and conducting an initial screening of titles and abstracts, we identified 84 relevant articles for further evaluation of their full texts. Out of these, 75 publications were excluded from the analysis due to reasons such as the absence of quantitative measures for relevant PA outcomes, qualitative studies, descriptive studies, literature reviews, commentaries, and others. Finally, our analysis included 8 longitudinal studies and 1 repeated cross-sectional study (10, 12, 16–18, 22, 29, 34, 35).

### 3.2 Characteristics of studies

Table 1 provides a summary of the characteristics of the nine studies analyzed in this research. The sample sizes varied between 169 and 1, 465 participants. The duration of the interventions ranged from 3 months to 30 months. Out of these studies, five (55.6%) were published since 2019 (10, 16–18, 34). In seven (77.8%) of the studies, the female participants exceeded the male participants (10, 16–18, 22, 29, 34). The included studies had sample populations from five countries, with four studies (44.5%) from North America (12, 16, 18, 22), two studies (22.2%) from Asia (10, 17), two studies (22.2%) from Europe (29, 34), and one study (11.1%) from Oceania (35). Out of the nine studies included in this analysis, five of them incorporated blue space (10, 17, 22, 29, 34). These studies used self-reported tools to assess PA. In total, more than five different self-reporting methods were utilized, including validated methods such as the Global PA Questionnaire (34) and non-validated questionnaires developed by researchers (12, 22).

**Table 1:** Characteristics of the included studies.

**Sources**	**Study design**	**Sample**	**Female (%)**	**Survey time**	**Exposure duration or completion date of the greenway**	**Intervention**	**Region**	**Outcomes and measures**
Merom et al. (35)	LS	450	44.9	2000.11; 2001.03	Three months	The construction of a 16.5-km-long Rail Trail cycleway and publicity	Australia	Walking, cycling (the 1999 National PA survey)
West and Shores (22)	LS	169	52.4	2007.12; 2008.12	Eleven months	5 miles of greenway were developed and added to an existing greenway along a river	USA	Walking, MPA, VPA (RDQ)
Goodman et al. (29)	LS	1,465	54.5	2010.04; 2012.04	Opened in September 2011.	Building or improvement of walking and cycling routes including two traffic-free bridges and an informal riverside footpath turned into a boardwalk	United Kingdom	Walking, cycling, all PA (IPAQ)
West and Shores (12)	LS	203	41.7	2009.11; 2011.11	One year	1.93 miles of greenway were developed and added to an existing greenway	USA	Walking, MPA, VPA (RDQ)
Frank et al. (16)	LS	524	55.3 (IG); 59.0 (CG)	2012.10–2013.03; 2014.10–2015.03	Opened in June 2013	The 2 km greenway is a major active transportation corridor	Canada	MVPA (IPAQ-SF)
Xie et al. (10)	LS	1,020	56.6	2016.04; 2019.04	Two and a half years	The East Lake greenway that was the original vehicle roads were converted into a 102-km-long greenway	China	MVPA, overall PA (IPAQ-SF12)
Frank et al. (18)	LS	524	55.3 (IG); 59.0 (CG)	2012.10–2013.03; 2014.10–2015.03	Opened in June 2013	The 2 km greenway is a major active transportation corridor	Canada	Cycling (a two-day travel diary)
He et al. (17)	LS	1,020	56.6	2016.04; 2019.04	Two and a half years	The East Lake greenway that was the original vehicle roads were converted into a 102-km-long greenway	China	Walking (IPAQ-SF12)
Hunter et al. (34)	Repeated CS	1,214	55.5	2010; 2017	Six months	Provision of a 9 km urban greenway along the course of 3 rivers and publicity	United Kingdom	Total PA (GPAQ)

All PA, overall PA, and total. PA, cumulative physical activity over the preceding seven-day period; CG, controlled group; CS, cross-sectional study; GPAQ, the Global Physical Activity Questionnaire; IG, intervention group; IPAQ, the International Physical Activity Questionnaire; IPAQ-SF, the short form International Physical Activity Questionnaire; LS, Longitudinal study; MVPA, moderate-to-vigorous intensity physical activity; MPA, moderate physical activity; PA, physical activity; RDQ, Researcher Developed Questionnaire; USA, the United States of America; VPA, vigorous physical activity.

### 3.3 Quality of the included studies

In this review, the NOS assessed eight longitudinal studies. The review included four high-quality studies with scores ranging between 7 and 8 (10, 16–18), as well as four low-quality studies with a score of 6 (12, 22, 29, 35). Supplementary Table S1 presents a comprehensive explanation of the NOS assessment process. Additionally, a repeated cross-sectional study was given a moderate-quality rating with an AHRQ score of 7 (34). Supplementary Table S2 contains information on the evaluation of quality.

### 3.4 Primary outcome

#### 3.4.1 Active travel

Six studies utilized questionnaires to report outcome indicators associated with AT, involving a total of 4, 081 participants (12, 17, 18, 22, 29, 35). The findings revealed that individuals residing in close proximity to the greenway exhibited a higher likelihood of experiencing improvements in their AT (SMD = 0.10, 95% CI: 0.04 to 0.17; I^2^ = 0.0%, *P*_*heterogeneity*_ = 0.49) (Table 2, Forest plot: Supplementary Figure S1). Additionally, the symmetrical funnel plot results and a *P*-value of 0.94 from the Egger regression test indicated the absence of significant publication bias (Supplementary Figure S2).

**Table 2:** Primary results based on various outcomes and subgroup analyses.

**Meta-analyses outcomes**	**Meta-analyses variables**	**No. of studies**	**No. of residents**		**Pool effect size**	**Heterogeneity**	
			**IG**	**CG**		**I**^2^ **(%)**	***P***
Primary outcome	AT	6	2,610	1,471	0.10 (0.04 to 0.17)	0.0	0.49
Secondary outcomes	MVPA	4	1,444	798	0.11 (0.02 to 0.20)	0.0	0.90
	Total PA	3	2,733	966	0.14 (0.06 to 0.21)	0.0	0.55
**Subgroup analysis based on the primary outcome of AT**							
Exposure duration	Overall	6	2,610	1,471	0.10 (0.04 to 0.17)	0.0	0.49
	Above or equal 12 months	3	1,135	606	0.11 (0.01 to 0.21)	0.0	0.86
	Below 12 months	3	1,475	865	0.10 (−0.03 to 0.23)	41.1	0.17
Male to female ratio	Overall	6	2,610	1,471	0.10 (0.04 to 0.17)	0.0	0.49
	Above or equal 1	2	513	393	0.09 (−0.13 to 0.31)	58.1	0.09
	Below 1	4	2,097	1,078	0.12 (0.04 to 0.19)	0.0	0.96
Exposure distance	Overall	6	2,610	1,471	0.10 (0.04 to 0.17)	0.0	0.49
	Within 1.00 km	2	332	358	0.08 (−0.07 to 0.23)	0.0	0.95
	1.00 km−2.00 km	4	2,278	1,113	0.11 (0.02 to 0.20)	24.8	0.26
Greenway characteristics	Overall	6	2,610	1,471	0.10 (0.04 to 0.17)	0.0	0.49
	Include blue space	3	1,858	793	0.12 (0.04 to 0.21)	0.0	0.96
	No blue space	3	752	678	0.08 (−0.06 to 0.22)	37.4	0.19
Total sample size	Overall	6	2,610	1,471	0.10 (0.04 to 0.17)	0.0	0.49
	Below or equal 450	3	606	466	0.09 (−0.08 to 0.25)	37.5	0.19
	Above 450	3	2,004	1,005	0.12 (0.04 to 0.20)	0.0	0.88
Region	Overall	6	2,610	1,471	0.10 (0.04 to 0.17)	0.0	0.49
	Europe and America	4	1,461	891	0.11 (0.02 to 0.19)	0.0	0.98
	China and Australia	2	1,149	580	0.11 (−0.07 to 0.29)	61.8	0.07

AT, Active Travel (walking and cycling for transportation); CG, controlled group; CI, confidence interval; IG, intervention group; MVPA, moderate-to-vigorous intensity physical activity (include moderate-intensity physical activity and vigorous-intensity physical activity); PA, physical activity; Pool effect size: pooled SMDs (95% CI); SMD, standard mean differences; Total PA: cumulative physical activity during the past seven-day period.

### 3.5 Secondary outcomes

#### 3.5.1 Moderate-to-vigorous PA

Four studies, with a total of 2, 242 participants, reported MVPA (10, 12, 16, 22). The analysis showed significant differences in MVPA (SMD = 0.11, 95% CI: 0.02 to 0.20; I^2^ = 0.0%, *P*_*heterogeneity*_ = 0.90) (Table 2, Forest plot: Supplementary Figure S3). The funnel plot demonstrated high symmetry, indicating the absence of publication bias. Additionally, Egger's test results (*P* = 0.63) suggested a low risk of publication bias (Supplementary Figure S4).

#### 3.5.2 Total PA

Three studies, involving a total of 3, 699 participants, reported on total PA (10, 29, 34). The analysis showed significant differences in total PA (SMD = 0.14, 95% CI: 0.06 to 0.21; I^2^ = 0.0%, *P*_*heterogeneity*_ = 0.55) (Table 2, Forest plot: Supplementary Figure S5). The funnel plot displayed high symmetry, suggesting the absence of publication bias. Additionally, the results of Egger's test (*P* = 0.49) indicated a minimal risk of publication bias in this analysis (Supplementary Figure S6).

### 3.6 Subgroup analyses

Subgroup analyses were conducted on the primary outcome measure of AT, using different variables of interest. The results indicated statistically significant differences among the subgroup items. For instance, participants who were exposed to the greenway for 12 months or longer (SMD = 0.11, 95% CI: 0.01 to 0.21, I^2^ = 0.0%, *P*_*heterogeneity*_ = 0.86) showed a significant improvement compared to those exposed for <12 months (SMD = 0.10, 95% CI: −0.03 to 0.23, I^2^ = 41.1%, *P*_*heterogeneity*_ = 0.17). Similarly, participants within the range of 1.00–2.00 km from the greenway (SMD = 0.11, 95% CI: 0.02 to 0.20, I^2^ = 24.8%, *P*_*heterogeneity*_ = 0.26) showed a similar result compared to those within 1.00 km from the greenway (SMD = 0.08, 95% CI: −0.07 to 0.23, I^2^ = 0.0%, *P*_*heterogeneity*_ = 0.95). The combined effect sizes for the subgroup analyses, calculated using the random-effects model, are presented in Table 2.

## 4 Discussion

To the best of our knowledge, this study represents the first comprehensive meta-analysis investigating the impact of greenway interventions on PA levels in participants. Our findings indicate a small but significant increase in PA levels among individuals residing near the greenway following the implementation of greenway interventions. Moreover, our results highlight that longer exposure to greenways, greenways incorporating blue space, intervention groups with a higher proportion of women, and participants living within a 2 km radius of a greenway experienced notable improvements in AT.

### 4.1 Main findings of the meta-analysis

This analysis suggests that greenway interventions have a positive impact on AT, MVPA, and total PA levels among nearby participants. This effect can be attributed to three primary factors. Firstly, the attractiveness of green spaces encourages individuals to engage in PA more frequently (36). Previous reviews support this view, indicating a strong association of 0.75 between green spaces and MVPA, highlighting the potential of landscape improvements to enhance the PA experience and promote PA (9, 15, 37). Secondly, greenways serve as linear infrastructure, connecting parks, open spaces, and public facilities, and have been shown in previous reviews to be associated with promoting PA and improving AT (37, 38). Furthermore, as traffic-calmed pathways, greenways enhance AT and promote PA by improving actual or perceived safety on the roads (39). Previous reviews have shown both positive and null associations between green space interventions, including greenways, parks, and similar interventions, and PA outcomes (13, 14). Our review demonstrates a substantial increase in PA among participants as a result of greenway interventions. This positive effect can be attributed to the distinctive spatial characteristics of greenways. The study highlights that individuals across all age groups show a preference for semi-natural green spaces over formal parks and sports fields (40). Greenways, due to their proximity to residential areas and provision of opportunities for walking in a semi-natural environment, are highly popular and greatly contribute to their utilization rates (40, 41). However, it is worth noting that certain studies have reported no significant rise in PA among participants residing near greenways (12, 34). This phenomenon can be attributed to a range of factors, including external influences such as social trends and psychological variables, as well as internal factors like the accessibility and openness of the greenways (34, 42). It is imperative to conduct further research to substantiate the existing findings, which should encompass comprehensive explanations of potential external and internal factors that may have a significant impact on the applicability of these findings to diverse urban areas. While this review primarily concentrates on individuals aged 16 and above, future studies should also explore the influence of greenways on PA among children and adolescents.

In the subgroup analysis of the primary outcome (AT) in this meta-analysis, statistically significant differences were observed within intervention effects in various subgroups. These groups included factors such as exposure duration, gender ratio, exposure distance, and greenway characteristics. Specially, when examining the subgroup based on exposure duration, participants with exposure duration exceeding 12 months exhibited a significant improvement in AT compared to those with exposure duration of less than 12 months. This finding suggests that longer greenway exposure time is associated with greater improvements in AT, because it takes time for behavior to settle (14). Shorter time periods are insufficient for accurately capturing habitual activity behaviors, as there is significant variability in weekly activity behaviors within individuals throughout the year and across different seasons (43). In order to assess the maintenance of behavior change, it is crucial to have a minimum exposure duration of 1 year (13). In addition, the effectiveness of interventions was found to be higher in populations with a greater proportion of females, which may be attributed to individuals' activity. Intercept surveys conducted with users of urban multiuse trails revealed that the majority of respondents reported utilizing the trails primarily for recreational activities (44). Recreational users in this study were found to cover longer distances and had a higher utilization rate of the trails. Surveys also indicated that females were more likely than males to visit the trails for leisure purposes, exercise, and to experience nature (44, 45). When considering intervention distance thresholds, it was discovered that effective thresholds fell within the range of 1.00 to 2.0 km, with distances <1.00 km being ineffective. In the studies, participants were divided into two groups: exposed and unexposed. This categorization was based on the proximity of their homes to green spaces, which was crucial for evaluating the effectiveness of the green space intervention. Previous research has indicated that individuals living near green spaces are more likely to engage in PA (46). However, there may be a threshold beyond which the distance to the green space starts to affect behaviors such as walking (47). This threshold is typically considered to be ~1.20 to 1.60 km, which is equivalent to about a 15-min walk, and is commonly referred to as a rule of thumb in walkability literature. On the other hand, other studies have found that most participants tend to frequently visit green spaces within 2.0 km of their homes (45, 48). The study outcomes may be influenced by thresholds below 1.00 km or exceeding 2.00 km, as participants may experience similar effects from green spaces. It is important to note that distance thresholds can vary depending on factors such as the type and size of the green space, cultural and social context, and the specific domain of PA. Hence, depending on the context, a range of 1.00–2.00 km may be appropriate for identifying exposed and unexposed groups. However, it is essential to emphasize that some studies have found no association between PA and the objective distance to green spaces (49), suggesting that objective distance may not be the most suitable indicator when exploring the relationship between PA and green space. Careful consideration should be given to selecting thresholds in future studies on greenspace interventions. Subgroup analysis revealed that greenways with water had larger intervention effects compared to those without water. Water is widely recognized as an important and attractive landscape element, and people generally prefer areas with water sources. It has been observed that natural scenes with water have a more positive impact on preference and rating judgments (50). Additionally, previous research has found positive associations between water features such as lakes and streams and PA in green spaces (49). Therefore, the study suggests that improving green corridors along canals could be an effective approach to increasing greenway usage and promoting PA (51). Consequently, incorporating water features in greenways may prove to be an effective intervention strategy for promoting PA among participants.

### 4.2 Strength and limitations

Our systematic review conducted a quantitative analysis to investigate the association between greenway interventions and participants' levels of PA. The main strength of our review lies in its emphasis on studies that evaluated PA levels before and after the greenway intervention, offering evidence to support a causal relationship between the greenway intervention and PA. No significant heterogeneity was observed, indicating that the effect size was representative of the overall population. However, this study did have some limitations that were identified. Firstly, the meta-analysis utilized outcome indicators from questionnaires, which may introduce recall bias or social desirability bias. Secondly, the majority of the studies included in this paper relied on natural experiments. While natural experiments are considered reliable and practical for studying the causal effects of the built environment on PA (52), a recent review assessing the risk of bias in natural experiments highlighted certain methodological limitations in key bias domains (53). Thirdly, the studies included in this review used various methods to select participants, assess greenway exposures, and measure outcomes. This diversity in methods could potentially introduce bias in the pooled estimates. Additionally, the studies in this review collected data on overall PA rather than specifically focusing on PA associated with greenways, which could also bias the findings. Therefore, it is important to exercise caution when interpreting the findings of our study, considering the aforementioned limitations.

### 4.3 Implications

In our research, we found that the greenway intervention had a small effect size on participants' PA, ranging from d = 0.10 to d = 0.14 (54). However, it is important to consider the impact of this effect size in real-world settings, which is influenced by participants' baseline PA levels. Previous reviews have demonstrated that effect sizes of d = 0.19 and d = 0.18 correspond to a weekly increase in PA duration of 15 and 73 minutes, respectively, based on participants' activity levels prior to the intervention (55, 56). The impact of even small effects on public health should not be underestimated, particularly when considering the cumulative effect over time and across large populations (57). Research indicates that even minor increases in PA resulting from the greenway intervention can lead to substantial health and cost benefits at the population level, as well as broader societal advantages (58). Therefore, caution is advised when interpreting the reported effects of the intervention.

Residents residing near a greenway exhibit a higher likelihood of participating in AT and engaging in MVPA. Such participation not only enhances physical fitness and reduces sedentary behavior but also fosters both physical and mental wellbeing. In order to encourage residents, particularly those living within a 2 km radius of a greenway, to increase their utilization and awareness of these pathways, it is recommended to implement initiatives like publicity campaigns, educational programs, and other related activities. These efforts will contribute to elevating PA levels within the community population. From a perspective of green space planning and design, it is imperative to enhance the accessibility of greenways, optimize their placement, incorporate them with blue spaces, and take into account the diverse preferences of residents. Considering the enduring beneficial effects of greenways on PA, local governments should give priority to their construction, refurnishment, and upkeep in urban green space planning to enhance public engagement and utilization. Furthermore, recent analyses on social return on investment have underscored the potential for greenways to generate positive economic returns (59, 60). Hence, the implementation of greenways for PA, encompassing their design, construction, and sustainable maintenance, emerges as a financially viable approach. Ultimately, greenways, when integrated into a comprehensive transportation and environmental system, possess substantial capacity to foster personal and communal wellbeing while also facilitating sustainable urban progress.

## 5 Conclusions

In this systematic review with meta-analysis, we present the most recent evidence indicating a small but meaningful increase in PA among individuals living near greenways. Moreover, subgroup analyses reveal that the impact of greenway interventions differs depending on specific moderating factors and environmental conditions. By objectively synthesizing existing research on greenway interventions and PA, this review offers valuable insights into the effects of green spaces on PA, highlighting the potential of greenways in promoting public health. Based on these findings, it is recommended that city managers and policymakers include greenways in their overall green space strategy, recognizing their construction and management as a crucial intervention for promoting public health. However, this review also highlights some limitations in current research designs. To improve the quality and accuracy of future studies, researchers in this field should strengthen the rigor of their experimental methods, concentrate on specific types of PA, and utilize advanced analytical techniques such as machine learning to reveal the intricate dynamics of greenway utilization.

## Data availability statement

The original contributions presented in the study are included in the article/Supplementary material, further inquiries can be directed to the corresponding authors.

## Author contributions

YD: Conceptualization, Data curation, Formal analysis, Investigation, Methodology, Project administration, Resources, Software, Validation, Writing – original draft. JL: Data curation, Formal analysis, Investigation, Methodology, Resources, Software, Validation, Writing – review & editing. QC: Conceptualization, Methodology, Supervision, Validation, Writing – review & editing.
