# Supplementary figures and images for: Greenway interventions effectively enhance physical activity levels—A systematic review with meta-analysis
Source: Front Public Health. 2023 Dec 6;11:1268502. doi: 10.3389/fpubh.2023.1268502 (PMC10745803; doi:10.3389/fpubh.2023.1268502)

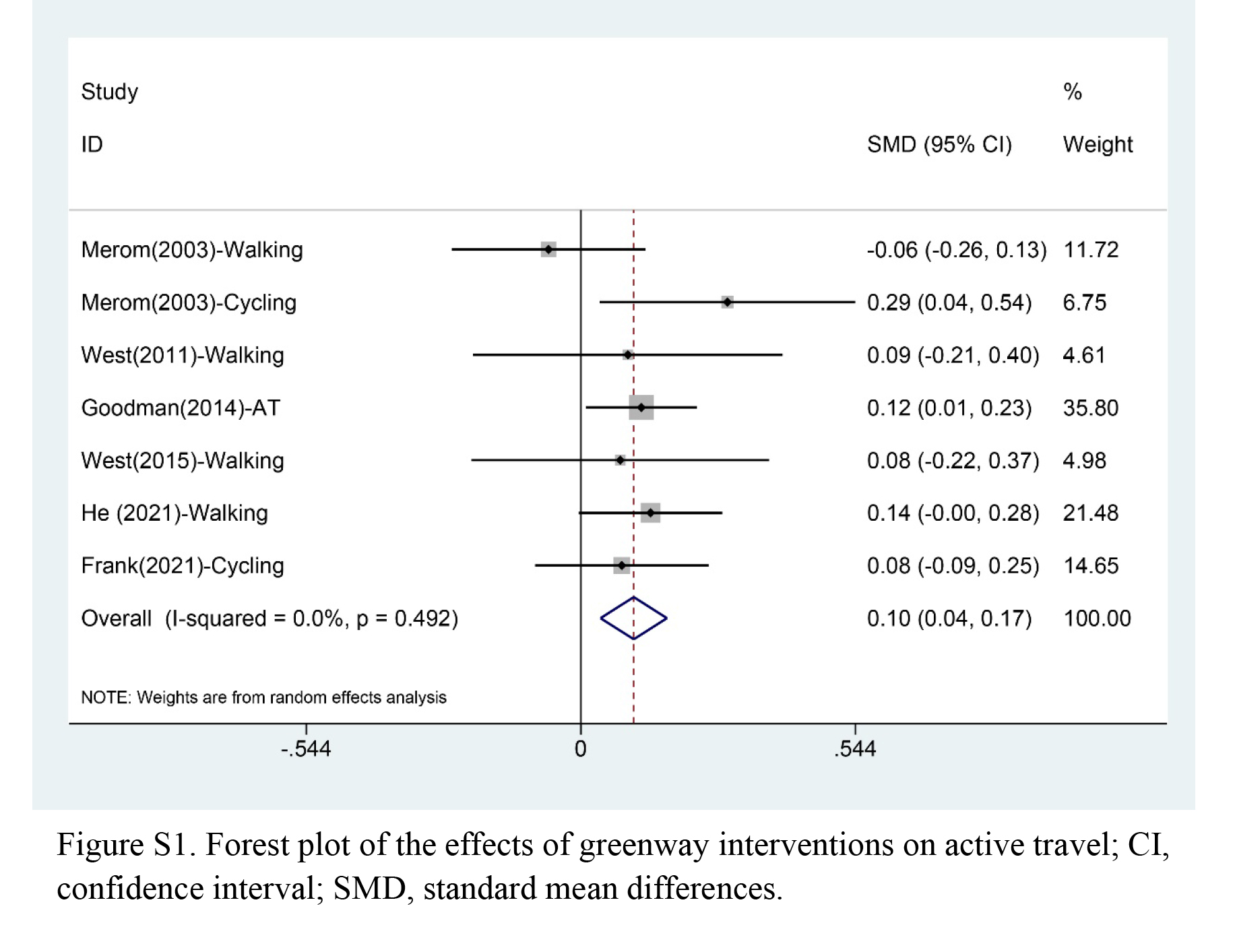

Supplement: Supplementary file 2 [file Image_1.JPEG]

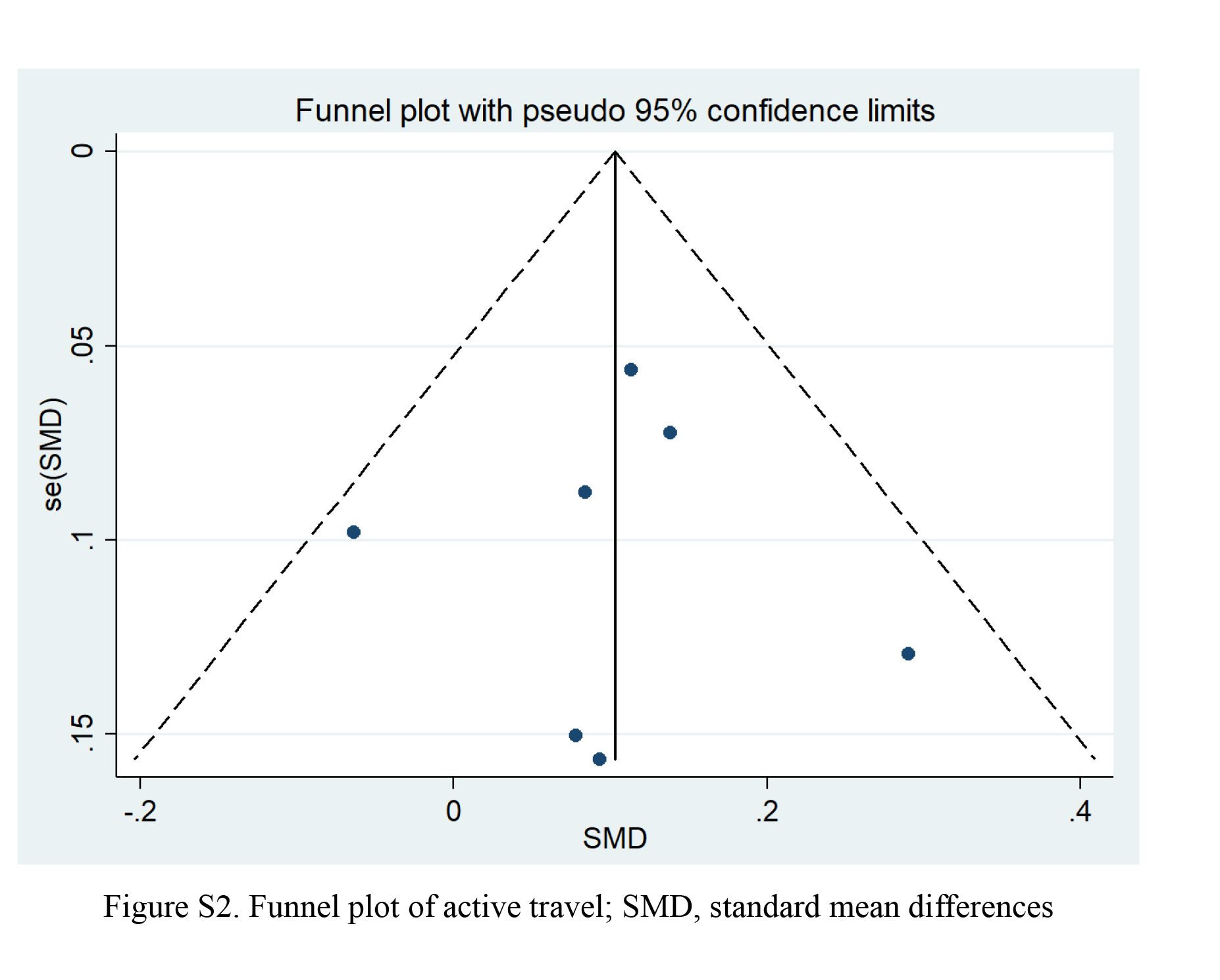

Supplement: Supplementary file 3 [file Image_2.JPEG]

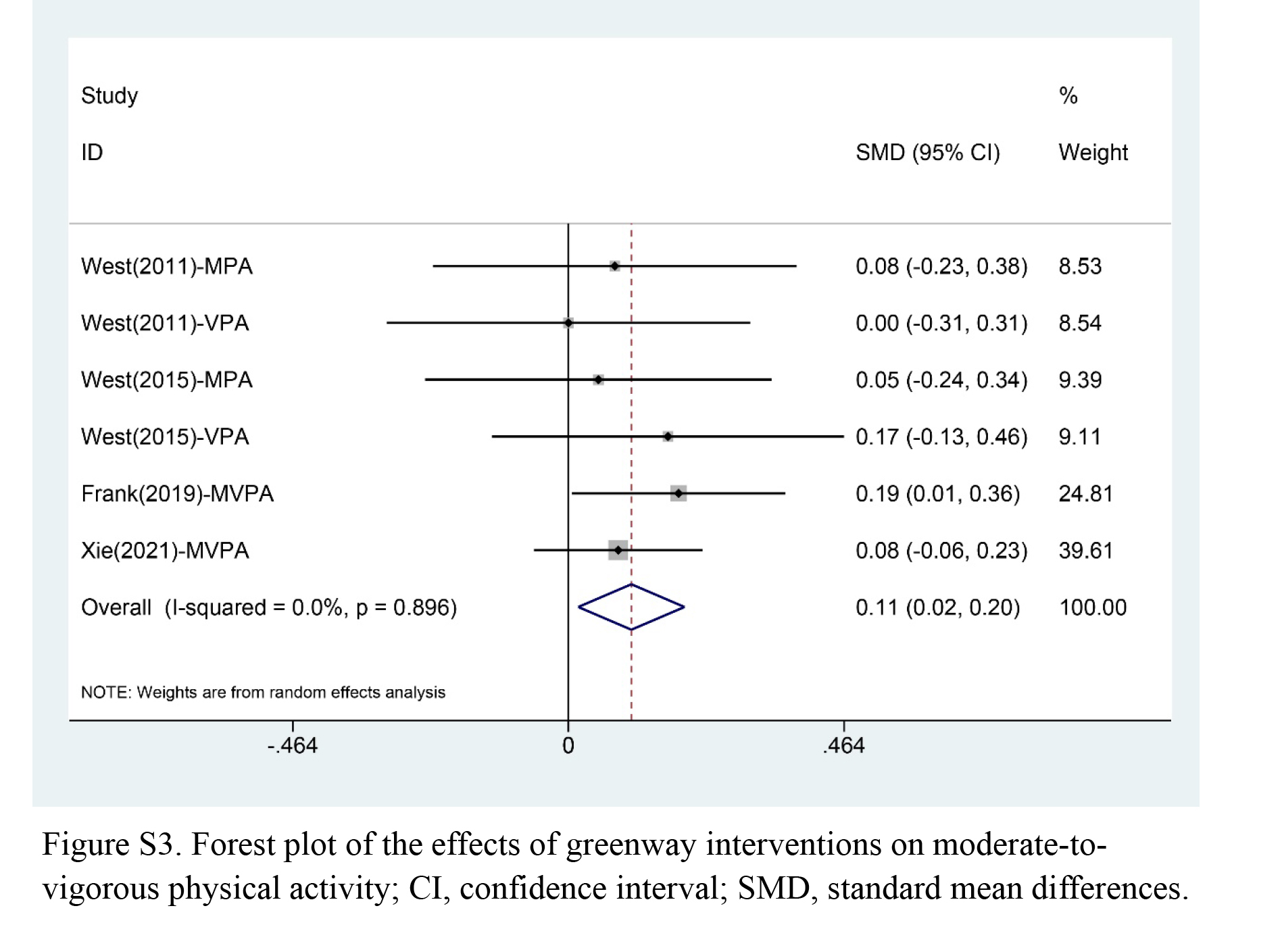

Supplement: Supplementary file 4 [file Image_3.JPEG]

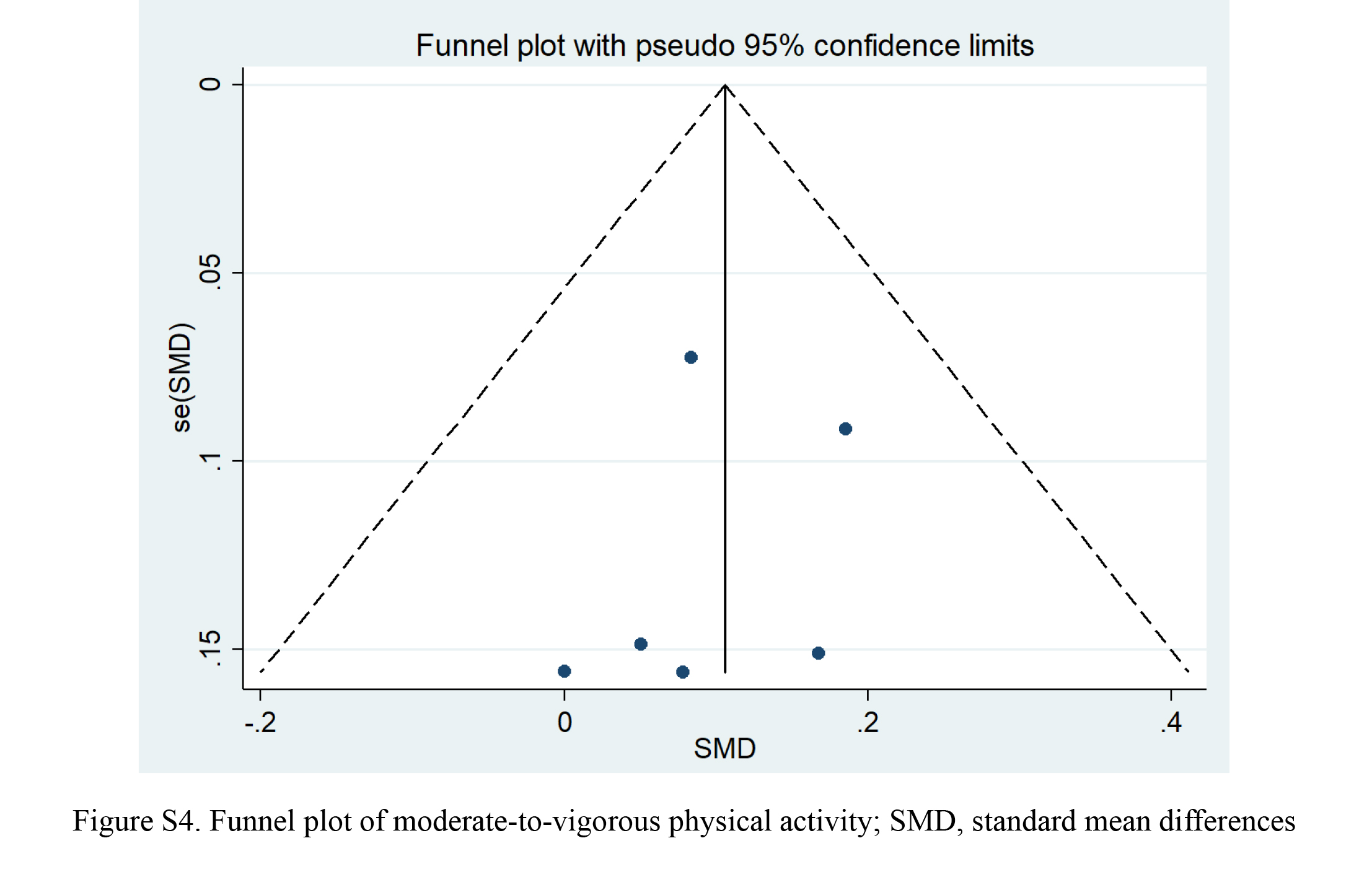

Supplement: Supplementary file 5 [file Image_4.JPEG]

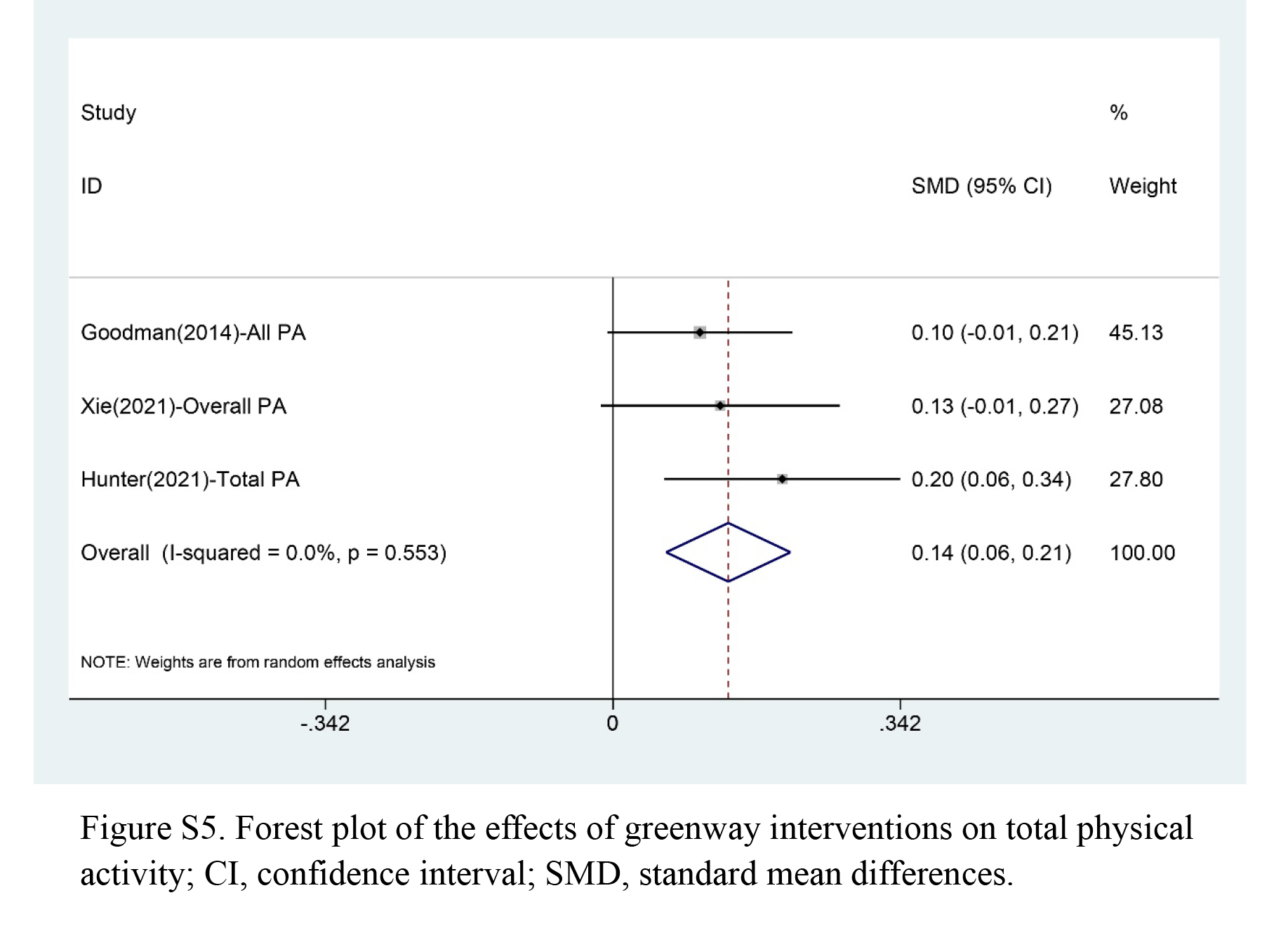

Supplement: Supplementary file 6 [file Image_5.JPEG]

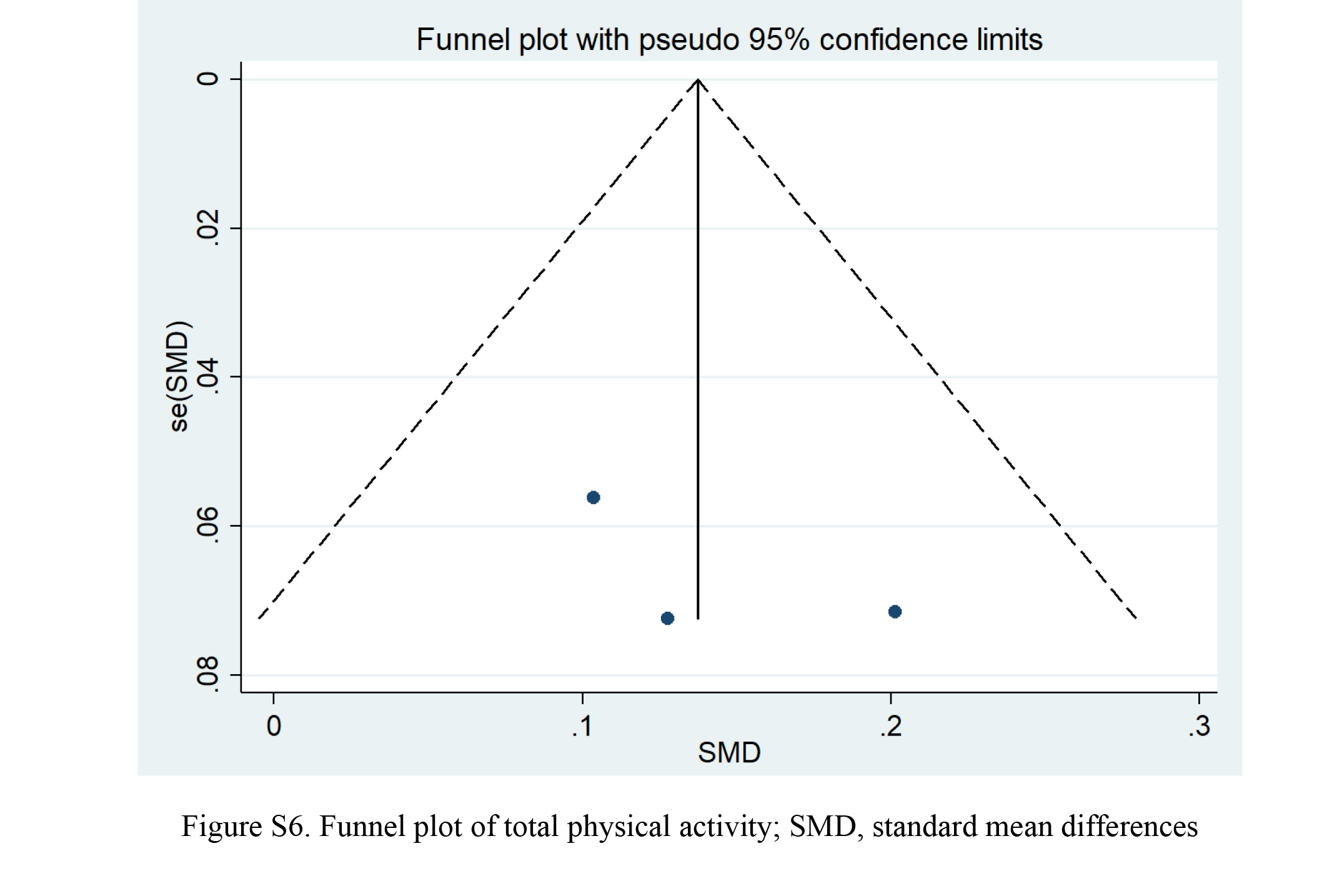

Supplement: Supplementary file 7 [file Image_6.JPEG]
